# Big data challenges in overcoming China’s water and air pollution: relevant data and indicators

**DOI:** 10.1007/s42452-021-04448-0

**Published:** 2021-03-18

**Authors:** Bo Zhang, Robert M. Hughes, Wayne S. Davis, Cong Cao

**Affiliations:** 1 Information Center, Ministry of Ecology and Environment, Beijing, China; 2 Amnis Opes Institute, Corvallis, OR USA; 3 Department of Fisheries and Wildlife, Oregon State University, Corvallis, OR USA; 4 USEPA (Retired), Washington, DC USA; 5 University of Nottingham, Ningbo, China

**Keywords:** Indicators, Monitoring, Assessment, Data quality, Pollution management

## Abstract

Big data are potentially useful for environmental management planning and actions that can be directed toward pollution control. China is using big data approaches to help reduce its current levels of pollution. However, also needed are better environmental indicators, measurement technologies, data management and reporting, and adaptive management and enforcement. Based on continental-extent monitoring and assessment programs in Europe and the USA, we recommend three major programmatic changes for China. (1) Establish long-term systemic environmental and human health objectives and indicators. (2) Adopt national standard methods for survey designs, sampling and analytical protocols, statistical analyses, and collaborative sampling programs. (3) Provide a transparent process for reporting and correcting data errors.

## Introduction

Recently, China has made considerable progress towards ecological and environmental protection through use of big data sets and analytics, which are increasingly being used by China’s Ministry of Ecology and Environment (MEE). The MEE is currently gathering ecological and environmental information into one map via a virtual set of map overlays [54]. Key aims include centralizing data management, promoting system integration, maximizing data transparency, and improving standards and data security. Most USA federal agencies responsible for environmental protection are also using big data sets and analytics in their work (e.g., [29, 89]).

There are 5 key challenges to China’s big data initiative: indicators, monitoring and assessment, data quality, data transparency, and rigorous adaptive management. If China can meet those challenges, it can fill substantial ecological research and management gaps by markedly increasing the extent and rigor of ecological information and pollution mitigation that is currently lacking in Asia [18]. Therefore, our goal in this paper is to provide specific examples of interdisciplinary and transdisciplinary big data programs that have been successfully implemented elsewhere.

### Quantitative indicators

**Fig. 1:**
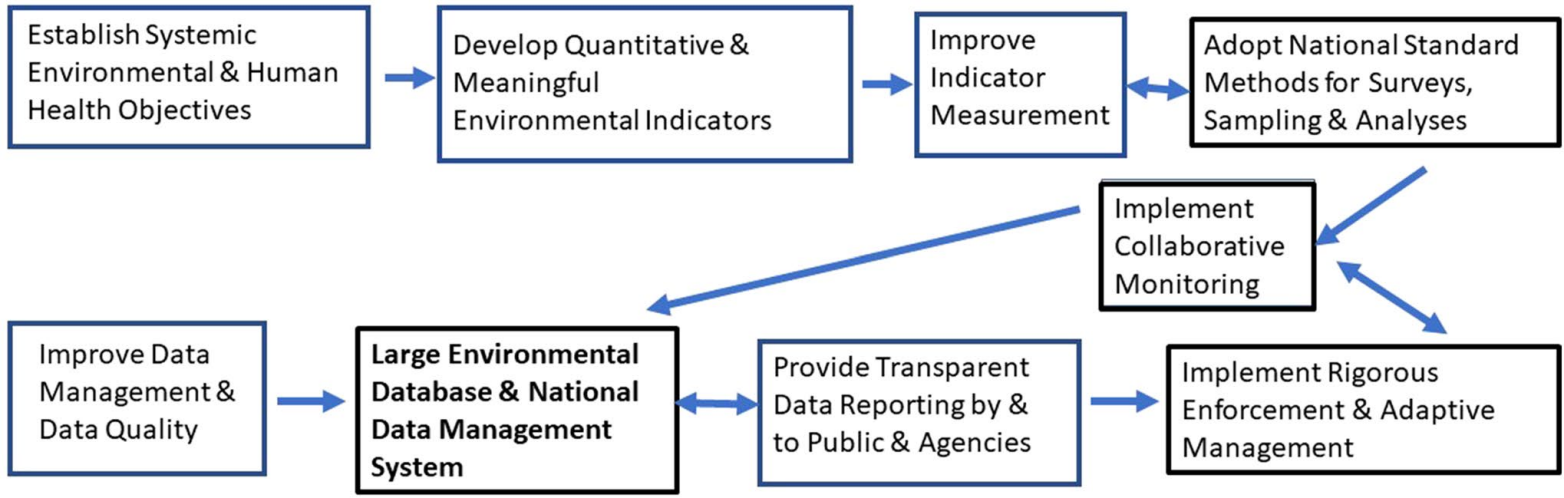
Although implementing a big data program is a goal for China, additional improvements are needed to ensure that the public is clearly informed and that environmental monitoring and enforcement are rigorously implemented, as indicated in the critical steps above

**Fig. 2:**
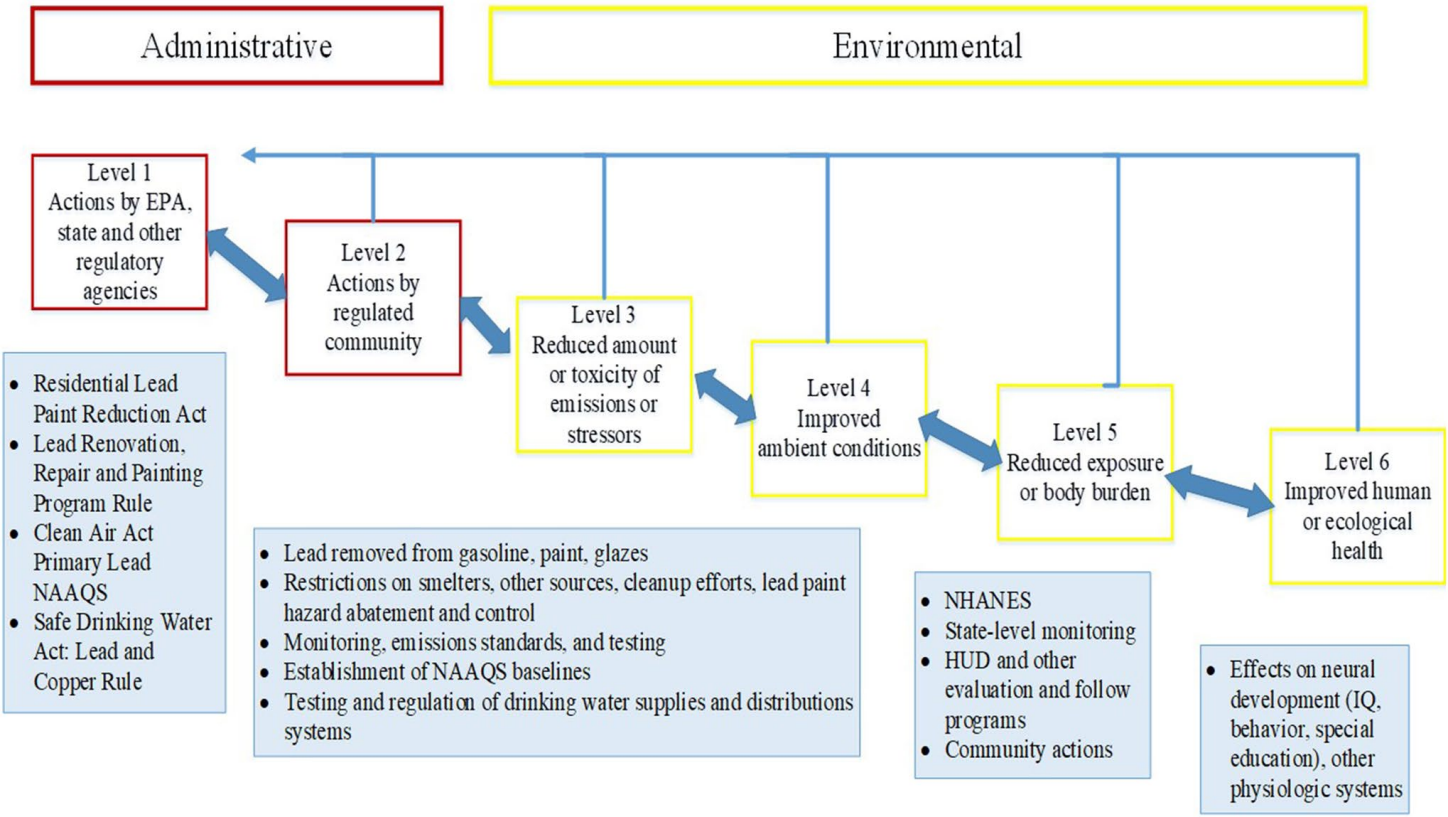
Administrative and environmental indicators: levels one through six

A suite of relevant, quantitative and cost-effective indicators is needed for tracking progress toward meeting clearly stated goals and objectives (Fig. 1; [34]). As shown in the figure, indicators begin with objectives or goals. For example, the goal of the USA Clean Air Act is to enhance “the quality of the Nation’s air resources so as to promote the public health and welfare” [72]. The objective of the USA Clean Water Act is to restore “the chemical, physical, and biological integrity of the nation’s waters” [73]. Subsequently, the U.S. Environmental Protection Agency (USEPA) developed a hierarchy of administrative and environmental indicators represented by six levels (Fig. 2; [74]). Levels 1 and 2 are administrative measures to be linked to improvements in Level 5 and 6 indicators that are related to human and ecological health.

For example, USEPA’s Level 5 and 6 indicators for surface waters include water quality, physical habitat structure, human health and biological assemblage indicators (Table 1). All those indicators were developed and tested in regional pilot studies before being implemented nationwide. Before being deemed acceptable, each biotic indicator was evaluated for its range of variation, sensitivity to anthropogenic disturbance, sampling error or reproducibility, redundancy with other candidate indicators and then adjusted for natural variability if needed [35, 45, 51, 53, 61, 71, 93]. The choices of water quality indicators were based on sampling ease and cost, shipping restrictions, widespread use by state and federal water quality agencies, and responsiveness to a wide range of anthropogenic stressors and pressures [27, 34, 93]. Physical habitat condition indicators were selected based on sampling ease, equipment cost and responsiveness to a wide range of anthropogenic stressors and pressures [5, 34, 41–43, 63]. Selection of human health indicators were based largely on their frequency of occurrence and known toxicities [58, 59]. These fish consumption indicators are used by USEPA to evaluate and reduce exposure to harmful water pollutants (e.g., [69, 91]). All the indicators listed in Table 1 are sampled by field crews using standard methods [34, 57, 76, 78, 79] at approximately 1000 sites every 5 years. Those assemblage sampling methods are based on rigorous sampling effort studies (e.g., [6, 33, 48, 62]). The sites are selected by use of a dispersed probability design to ensure statistical representativeness, minimize site proximity and ensure rigorous status and trend assessments [56, 70].

**Table 1:** Physical, chemical, and biological indicators of the USEPA national rivers and streams assessment, and the percent of waters in good and poor condition nationally. China lacks national monitoring of any of the listed indicators—except for total P & total N (from USEPA [86])

Class	Indicators
Biotic condition	Fish assemblage multimetric index 26% good; 37% poor
	Macroinvertebrate assemblage multimetric index 30% good; 44% poor
Water quality	Total phosphorus 18% good; 58% poor
	Total nitrogen 32% good; 43% poor
	Salinity 86% good; 4% poor
	Acid neutralizing capacity 98% good; 1% poor
Physical habitat structure	Excess fine sediments 52% good; 22% poor
	Fish habitat condition 64% good; 14% poor
	Riparian vegetation cover 58% good; 24% poor
	Anthropogenic riparian disturbance 29% good; 23% poor
Human health	River fish tissue Hg 24% poor
	Urban river fish tissue PFOs 3% poor
	Urban river fish tissue PCBs 40% poor

USEPA’s Level 5 and 6 air quality indicators include several of those monitored in China (Table 2), plus many additional toxic metals and organics as well as an air quality index based on 7 widespread contaminants (ground-level O_3_, 2.5 µ and 10 µ particulates, Pb, CO, SO_x_, NO_x_). Those indicators were selected because of their known correlations with the incidence of respiratory, cardiac and cancerous diseases [88]. They are monitored at approximately 3900 stations. Ambient air quality monitoring for toxic and non-toxic, or criteria, pollutants is primarily the responsibility of State agencies in the USA. EPA’s Reports on the Environment (ROEs) are updated online reports available to the public [80], which include human exposure, health, and other indicators linked with ambient air indicators to reduce exposure to harmful air pollutants.

**Table 2:** Key air pollutants monitored nationally and by source in China and the USA [38, 84, 85, 87]. Criteria successes are based on 90th percentiles of USA averages for ambient concentrations

China	USA
Total SO_2_ emissions	Total anthropogenic SO_2_ emissions Met criteria since 2002
Total NO_2_ emissions	Total NO_2_ emissions Met criteria since 2006
Total NO_x_ emissions	Total anthropogenic NO_x_ emissions Met criteria since 1980
Total O_3_ emissions	Total anthropogenic O_3_ emissions Met criteria since 2013
Total CO emissions	Total anthropogenic CO emissions Met criteria since 1992
Total PM2.5 emissions	Total anthropogenic PM2.5 emissions Met criteria since 2010
Total PM10 emissions	Total anthropogenic PM10 emissions Met criteria since 1991
–	Total anthropogenic As emissions
–	Total anthropogenic Cd emissions
–	Total anthropogenic Cr emissions
–	Total anthropogenic Hg emissions Met criteria since 2015
–	Total anthropogenic Pb emissions Met criteria since 2006
–	Total air toxic (187 pollutants) emissions
–	Air Quality Index

USEPA’s water monitoring and regulation programs have two key limitations. Results have indicated that key anthropogenic pressures are largely related to minimally regulated diffuse pollution [18, 40, 46] compared with point source pollution, which has been widely curtailed. That pollution originates from agriculture [9, 30, 44, 75], livestock grazing [1, 19], mining [11, 37, 95], flow regime alteration [36, 60] and non-native invasive species [36, 49]. Furthermore, such pressures are frequently co-occurring both in the USA [4, 7] and Europe [66].

Regarding USA air pollution monitoring and regulation, there are 5 shortcomings. A growing concern is USEPA’s failure to maintain its automated air monitoring stations [52]. Also, criteria air pollutants are sampled non-randomly near suspected sources, the data sources and modeling methods vary, and they are presented at such a granular level as to hinder statistically rigorous national statements.

The European Union (EU) approved its Water Framework Directive in 2000, with a goal of good water quality by protecting all types of water bodies, restoring ecosystems, reducing pollution, and guaranteeing sustainable water usage [16]. Good ecosystem condition is defined by use of fish, macroinvertebrate, algae, and macrophyte assemblage indicators (e.g., [25]) together with hydromorphology and water quality indicators. The EU 2013 Clean Air Protection Programme is focused on reducing air pollution, especially in cities [15], setting stricter national emission ceilings for six major pollutants, and reducing pollution from medium-sized pollution generators. Current ambient air criteria cover 12 major pollutants with monitoring periods from 1 h to 1 year.

The Chinese objectives neglect information about the extent to which pollution damages the environment or human health. China measures administrative and environmental indicators involving Level 1–4 indicators such as the percentages of surface waters and airsheds meeting, or not meeting, acceptable ranges of key chemical constituents. Although increases in asthma and other chronic respiratory diseases and premature mortality occur throughout China from ambient air pollution [17, 24], Level 5 and 6 indicators are missing for quantitatively measuring the effects of air and water quality on human health and biota.

### Monitoring and assessment

China has implemented a network of ambient air quality monitors providing data that MEE and the provincial and local environmental protection bureaus (EPBs) can use to assess progress toward meeting established goals. However, many cases of monitoring system and data manipulation by local government officials have been reported, and several key air pollutants are still not monitored ([22, 98], Table 2).

Unlike water body monitoring in China, the USEPA implemented a National Aquatic Resources Survey (NARS) in 2000 based on a probabilistic design, standard sampling methods and indicators, and collaborative sampling by the states and government contractors. NARS provides statistically valid assessment of the ecological status and the relative importance of various stressors to all USA surface waters [77, 81–83, 86]. China has no similar national program.

Key results from the USEPA’s NARS indicate that 3–40% of the sampled population of rivers contain fish tissue levels of mercury, polychlorinated biphenyls or perfluorooctanesulfunic acid that warrant fish consumption warnings (Table 1, [86]). Regarding physical habitat structure, 14–24% of surface waters are in poor condition for fish habitat condition, excess fine sediments, riparian disturbance or riparian vegetation condition. Nutrient levels indicate that 43–58% of stream length is in poor condition. Based on the assemblage condition of fish and macroinvertebrates 37% and 44% of stream and river length is in poor condition (Table 1; [86]).

EPA’s Report on the Environment [85] indicated that each of the criteria air pollutants has decreased over time and have met national standards for 5–20 years (Table 2). Although the National Air Toxics Assessment (NATA) reports are only published about every 4 years, there is a source of consistent and comparable data regarding air toxics that is part of the annual Toxics Release Inventory, a self-reporting system of the most significant industrial sources of pollution. A similar exposure modeling routine as used in NATA, called Risk Screening Environmental Indicators (RSEI), is employed to estimate ambient concentrations of air toxics with the goal of establishing the relative human health risks throughout the USA. RSEI uses a big data analytics application, called Qlik Sense, to display national data with the ability to sift the results down to a single facility among the over 20,000 facilities in the database [87]. The RSEI national relative risk trend decreased since 2007 and has remained relatively stable since 2016. However, the RSEI model results indicate continued risks to economically disadvantaged populations [10, 97], which also indicates the value of publicly available pollution data.

The EU member states use ad hoc approaches to select sites, but many states employ standard field methods and data analyses and measure multiple assemblages at least annually at each site. Those differences in methods and data interpretation complicate some EU-wide assessments [26] because observed differences in assemblages are confounded by differences in field sampling and data processing protocols. But see [64, 65] for examples obtained from standard sampling methods. Schinegger et al. [66] reported that 73% of 3105 study sites were impaired; 43% of all sites were impaired by multiple stressors.

Citizen-science and consumer-based environmental monitoring equipment has made it easier for citizens to participate in data collection and scientific research [12]. Such citizens have measured water quality, assessed physical habitat conditions, and evaluated fish and macroinvertebrate status through use of standard protocols (e.g., [20, 55]. Citizen scientists are typically more invested in environmental improvement and protection than the average citizen [20, 21].

It would be cost-effective for China to adopt national standard methods for survey designs, sampling and analytical protocols, statistical analyses, collaborative sampling by provincial governments and academics, open data reporting, and peer-review publications. Doing so could engage an enormous number of scientific collaborators as well as markedly increase database sizes and quality, thereby improving data quality and usefulness. For example, in Oregon (USA), water body monitoring and assessment data from eight different institutions were easily synthesized because all used a standard survey design, standard methods, and the same environmental and biological indicators [55]. Multiple Brazilian universities employed the same NARS survey design, standard sampling methods, and indicators for regionally assessing Atlantic Forest [39] Cerrado [8, 68] and Amazônia [3, 47] streams. Jimenez-Valencia et al. [39] determined that 62% of basin stream length was in poor condition based on macroinvertebrate assemblage condition,the risk of poor condition was four-fold greater in catchments with degraded forest. Silva et al. [67] estimated that 27% of the stream length in four hydrologic units had poor macroinvertebrate condition. Those poor conditions were twice as likely at high levels of turbidity and fine sediments [68]. Leitão et al. [47] reported that local and catchment deforestation decreased instream large wood, which decreased fish species richness and functional originality. Such assessments would have been inconceivable by single institutions working alone and using differing protocols.

### Data quality

Problems exist in China with data availability, interruptions in time series, inconsistencies between different sources reporting similar energy and air quality statistics, and a lack of data transparency [31, 38]. China’s pollution emissions data have not been standardized, leading to uncertainty in results and preventing EPBs from fully assessing actual discharge conditions [90]. In comparison, EPA and EU data quality from monitoring and assessment programs allow continental-extent assessments and scientific interpretations of key causal factors for all USA (e.g., [77, 81–83, 86]) and many EU [23, 65] surface waters. EU indicators differ somewhat among nations, but they have been intercalibrated for making Europe-wide assessments [23, 65]. Both standard methods and a rigorous statistical probability design allow inference to all conterminous USA surface waters with known confidence intervals [77, 81–83, 86].

We offer five recommendations for improving China’s data quality based on USEPA [84]. (1) All data must contain metadata for interpreting and reproducing results to ensure that all potential users can analyze the data. (2) A single centralized data exchange should be implemented, in which the different participants maintain their own databases but seamlessly exchange a common set of necessary data for tracking national, provincial, and local environmental progress. (3) Require that enterprises, MEE, and EPBs electronically report quantitative pollution discharge data to the central data exchange. (4) Implement effective supervision and auditing through periodic, unscheduled inspections of enterprises, MEE, and EPBs to verify their reporting to correct data errors immediately. (5) Impose stiff penalties and public notice for individuals and entities that deliberately or accidentally falsify data and hold governmental officials accountable for any environmental data fraud cases under their jurisdiction.

### Data transparency

The transparency of China’s environmental data remains problematic [14]. Data are scattered across various platforms and agency websites. The disclosure of information from EIAs is insufficient to support meaningful and robust public engagement. Conversely, in the USA, the NARS data are available for non-federal users to download, analyze, interpret, and publish as they wish, which greatly amplifies the usefulness and applications of the data (e.g., [9, 13, 28]). EPA electronic reporting and disclosure of large databases includes water pollution discharge monitoring reports and industry self-reported toxics release inventories. USEPA’s [75] information quality guidelines provide a process for the public to access and officially report errors they find in the data from almost 22,000 facilities and to have them corrected.

### Adaptive management

We believe that a key towards continued improvement requires government employees to view themselves as ecosystem (nature) trustees always keeping in mind the goals and objectives of the empowering legislation [94]. Citizens should be viewed as the trust beneficiaries, nature should be viewed as quantifiably valued assets of the trust, and government decision-making should be based on the fiduciary responsibility of the trustee. Polluters should be viewed as nature and human despoilers, their payments to government employees should be viewed as illegal bribes, and government employees who fail to meet their fiduciary responsibilities should be treated as criminal offenders [92, 94].

Resource management should be based on a rigorous adaptive management paradigm [50, 96]. Rigorous adaptive management and plans are based on: (1) specifying clear sets of general goals and explicit objectives; (2) explicitly stating actions that will and will not be taken when pre-identified trigger points occur; (3) explicitly identifying what is and is not known about the problems and their resolution; (4) clearly explaining expected conditions; (5) designing and implementing monitoring programs for learning about the above uncertainties; and (5) using the monitoring information to revise predictive models and management actions.

## Conclusions

Common concerns to pollution control globally include government inaction, resistance of industries and citizens toward environmental protection, false arguments of jobs versus the environment and human health, externalities and lag effects, and resistance toward long-term and large-extent strategic thinking. But those also are the drivers for better analysis of big data. China can draw on international and historical experience in tackling its environmental challenges and improving its big data. The implementation of such an initiative goes beyond the technology itself. It requires the efforts of the entire society, the resolve of political leaders, and an institutional framework to fully understand the indicators developed from the data and how the country will most effectively use the data. The successes or failures that China exhibits in resolving its air and water pollution problems will serve as explicit indicators of its ability to solve environmental problems affecting human health and quality of life nationally and globally.
